# Lipid Inhibitory Effect of (−)-loliolide Isolated from *Sargassum horneri* in 3T3-L1 Adipocytes: Inhibitory Mechanism of Adipose-Specific Proteins

**DOI:** 10.3390/md19020096

**Published:** 2021-02-08

**Authors:** Hyo-Geun Lee, Hyun-Soo Kim, Jun-Geon Je, Jin Hwang, K. K. Asanka Sanjeewa, Dae-Sung Lee, Kyung-Mo Song, Yun-Sang Choi, Min-Cheol Kang, You-Jin Jeon

**Affiliations:** 1 Department of Marine Life Science, Jeju National University, Jeju 63243, Korea; hyogeunlee92@jejunu.ac.kr (H.-G.L.); wpwnsrjs3@jejunu.ac.kr (J.-G.J.); ghkdwls9280@jejunu.ac.kr (J.H.); asanka@jejunu.ac.kr (K.K.A.S.); 2 Marine Biodiversity Institute of Korea, 75, Jangsan-ro 101-gil, Janghang-eup, Seocheon 33362, Korea; gustn783@mabik.re.kr (H.-S.K.); daesung@mabik.re.kr (D.-S.L.); 3 Research Group of Food Processing, Korea Food Research Institute, 245, Nongsaengmyeong-ro, Iseo-myeon, Wanju 55365, Korea; rudah@kfri.re.kr (K.-M.S.); kcys0517@kfri.re.kr (Y.-S.C.)

**Keywords:** *Sargassum horneri*, (−)-loliolide, lipid metabolism, 3T3-L1 adipocytes

## Abstract

*Sargassum horneri* (*S. horneri*) is a well-known brown seaweed widely distributed worldwide. Several biological activities of *S. horneri* have been reported. However, its effects on lipid metabolism and the underlying mechanisms remain elusive. In the present study, we examined the inhibitory effect of the active compound “(−)-loliolide ((6S,7aR)-6-hydroxy-4,4,7a-trimethyl-5,6,7,7a-tetrahydro-1-benzofuran-2(4H)-one (HTT))” from *S. horneri* extract on lipid accumulation in differentiated adipocytes. MTT assays demonstrated that (−)-loliolide is not toxic to 3T3-L1 adipocytes in a range of concentrations. (−)-loliolide significantly reduced intracellular lipid accumulation in the differentiated phase of 3T3-L1 adipocytes as shown by Oil Red O staining. Western blot analysis revealed that (−)-loliolide increased the expression of lipolytic protein phospho-hormone-sensitive lipase (p-HSL) and thermogenic protein peroxisome proliferator-activated receptor gamma coactivator 1-alpha (PGC-1). Additionally, (−)-loliolide decreased expression of adipogenic and lipogenic proteins, including sterol regulatory element-binding protein-1 (SREBP-1), peroxisome proliferator-activated receptor-γ (PPAR-γ), CCAAT/enhancer-binding protein-α (C/EBP-α), and fatty acid-binding protein 4 (FABP4) in 3T3-L1 adipocytes. These results indicate that (−)-loliolide from *S. horneri* could suppress lipid accumulation via regulation of antiadipogenic and prolipolytic mechanisms in 3T3-L1 cells. Considering the multifunctional effect of (−)-loliolide, it can be useful as a lipid-lowering agent in the management of patients who suffer from obesity.

## 1. Introduction

Due to its steadily increasing rates, obesity is regarded as a global epidemic and public health concern [1]. Based on statistics, more than 2.1 billion people are either overweight or suffer from obesity, and approximately 3.4 million obese patients die annually [2]. Pediatric and childhood obesity have increased in the last few decades, with obese children and adolescents accounting for approximately 21–24% of obesity patients [3]. Childhood obesity is closely related to adult obesity, develops more easily into adult obesity, and is accompanied by an increased risk of obesity-related diseases such as cancer and cardiovascular disease [4,5,6]. Indeed, childhood obesity and obesity-related comorbidities increase the incidence of cardiovascular disease and nonalcoholic fatty liver disease in children and adolescents [7]. Therefore, several guidelines and programs have been reported for the treatment of childhood obesity [8]. The World Health Organization (WHO) defines overweight and obesity as abnormal or excessive fat accumulation in the body, with a body mass index (BMI) over 30. The imbalance of energy expenditure and calorie intake in the human body can induce overweight or obesity. A number of complex interactions between genetics, behavior, and environmental factors affects the development of obesity [9]. Obese patients have high levels of triglyceride (TG), total cholesterol (TC), decreased high-density lipoprotein (HDL), and abnormal low-density lipoprotein (LDL) composition [10]. High TG and cholesterol levels can induce severe metabolic disorders [11]. Moreover, abnormally elevated TG and cholesterol levels increase the risk of several comorbidities such as diabetes mellitus, cardiovascular disease, and cancer [12]. Accordingly, obesity increases the risk of metabolic diseases. Han and Mike (2016) reported that the clinical perspective of obesity, metabolic syndrome, cardiovascular disease (CVD), and coronary heart diseases (CHD) and their symptoms can be improved by decreasing TG, TC, and HDL levels [13]. Long-lasting overweight conditions exacerbate adverse effects, such as cardiometabolic risk factors that induce cardiovascular diseases, including CHD, stroke, and diabetes mellitus [14]. Thus, commercial anti-obesity agents have been developed in the medicinal industry to treat patients who suffer from obesity. Currently, several kinds of commercial anti-obesity drugs have been developed worldwide [15]. Among these, orlistat (Xenical) and sibutramine (Meridia) are the most commonly used anti-obesity agents to treat obesity. Orlistat was approved by the U.S. Food and Drug Administration (FDA). In general, Xenical reduces fat absorption from the intestine via suppression of gastric and pancreatic lipase activity. This breaks down the TG and reduces fat accumulation in the body [16]. However, orlistat has some undesirable side effects such as oily bowel steatorrhea [17]. In addition, prolonged use of orlistat was also found to induce toxicity in the human pancreas and kidney [18]. Sibutramine deactivates neurotransmitters, which are chemical messengers between neurons and target cells. In turn, this blocks the reuptake of serotonin and norepinephrine and may increase serotonin levels that induce satiety and decrease appetite [19]. However, sibutramine also has various adverse effects, including headaches, insomnia, and amnesia [20]. Therefore, the development of new natural anti-obesity agents is an urgent requirement to treat obese patients without inducing side effects. Most studies on obesity have focused on the potential beneficial effects of natural products from land plants [21]. Furthermore, several publications have reported the excellent anti-obesity activities of marine-derived active compounds [22,23].

*Sargassum horneri* (*S. horneri*) belongs to the *Sargassum* genus, which is abundant in Korea and Japan. *S. horneri* extract has been used for medicinal purposes in traditional medicine [24]. In addition, the active component of *S. horneri* showed various biological properties, such as antioxidant, anti-inflammatory, anti-wrinkle, and immunomodulatory activities [25]. (−)-loliolide ((6S,7aR)-6-hydroxy-4,4,7a-trimethyl-5,6,7,7a-tetrahydro-1-benzofuran-2(4H)-one (HTT)) is composed of a series of pigment compounds and exhibits antioxidant, anti-apoptotic, and antiviral activity [26,27,28]. However, the inhibitory effects of (−)-loliolide from *S. horneri* on lipid accumulation have rarely been investigated. Kwon et al. (2019) investigated the lipid inhibitory effect of an ethanol extract separated from *S. horneri* on 3T3-L1 adipocytes [29]. In the present study, the inhibitory effects of (−)-loliolide on lipid accumulation were determined in differentiated 3T3-L1 adipocytes [30]. Furthermore, adipose-specific protein expression was determined to investigate the intracellular lipid inhibitory mechanisms in vitro.

## 2. Results

### 2.1. (−)-loliolide Is not Cytotoxic and Inhibits Lipid Accumulation in Differentiated 3T3-L1 Cells

**Figure 1:**
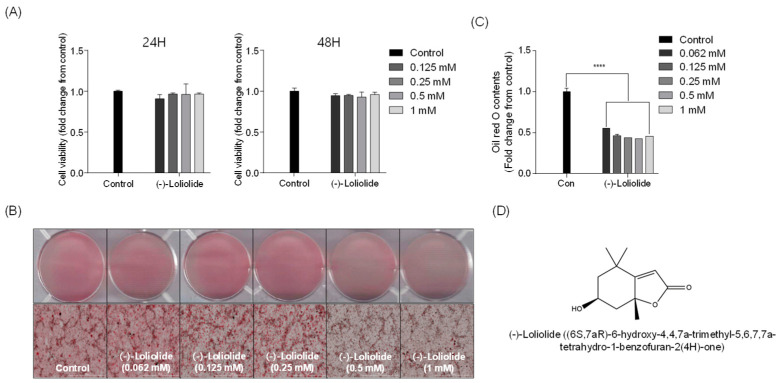
(−)-loliolide contrasts lipid accumulation in 3T3-L1 cells. (**A**) Cytotoxic effect of (−)-loliolide on cell viability in 3T3-L1 measured for 24 and 48 h. (**B**) Microscopic images of 3T3-L1 cells stained with Oil Red O (ORO) and (**C**) relative lipid accumulation. (**D**) The structure of (−)-loliolide. All data are presented as mean ± SD (*n* = 3). Significant differences were identified at **** *p* < 0.0001 compared to the control group.

The cytotoxicity of different concentrations of (−)-loliolide (0.125, 0.25, 0.5, and 1 mM) was investigated in 3T3-L1 cells (Figure 1A). At the tested range, (−)-loliolide did not show cytotoxicity in 3T3-L1 cells. Thus, these nontoxic concentrations were selected for further experiments. Next, differentiation of 3T3-L1 cells was induced to promote adipogenesis and lipid accumulation. Figure 1B shows the accumulation of lipids in 3T3-L1 cells. High lipid accumulation was observed in the control group (untreated samples). However, treatment with (−)-loliolide significantly decreased intracellular lipid accumulation in differentiated 3T3-L1 cells. A significant reduction in lipid accumulation was detected in the (−)-loliolide -treated group. The isolation and purification procedure of (−)-loliolide from *S. horneri* were kindly described by Kim et al. (2020) [31] and the structure of (−)-loliolide is represented in Figure 1D. These results indicate that supplementation with (−)-loliolide significantly suppressed lipid accumulation in 3T3-L1 adipocytes.

### 2.2. (−)-loliolide Suppresses Adipogenic and Lipogenic Pathways in 3T3-L1 Cells

**Figure 2:**
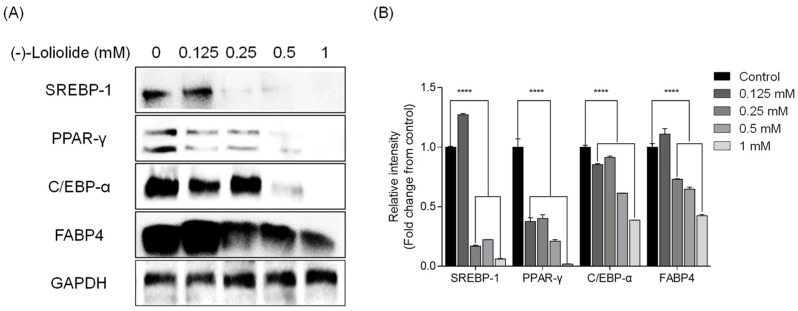
(−)-loliolide regulates adipogenesis and lipogenesis pathway enzyme expression in 3T3-L1 cells. (**A**) Western blot analysis of lipogenic SREBP-1 and adipogenic PPAR-γ, C/EBP-α, and FABP4. (**B**) Quantification graph for expression of SREBP-1, PPAR-γ, C/EBP-α, and FABP4. All data are presented as mean ± SD (*n* = 3). Significant differences were identified at **** *p* < 0.0001 compared to the control group.

Next, Western blot analysis was performed to elucidate the potential inhibitory effect of (−)-loliolide on expression of adipogenic and lipogenic proteins. The levels of adipogenic proteins peroxisome proliferator-activated receptor-γ (PPAR-γ), CCAAT/enhancer-binding protein-α (C/EBP-α), and fatty acid-binding protein 4 (FABP4) were increased in control cells, which were only treated to induce adipocyte differentiation [32]. However, adipogenic protein expression was lower in the presence of (−)-loliolide. In particular, the highest concentration of (−)-loliolide (1 mM) dramatically decreased the expression of the adipogenic proteins (Figure 2). In addition, the levels of lipogenic protein sterol regulatory element-binding protein-1 (SREBP-1) were significantly reduced following (−)-loliolide treatment. Taken together, these results suggest that (−)-loliolide strongly suppressed adipogenesis and lipogenesis by reducing expression of adipogenic and lipogenic proteins in 3T3-L1 cells. 

### 2.3. (−)-loliolide Regulates Thermogenesis and Lipolysis in 3T3-L1 Cells

**Figure 3:**
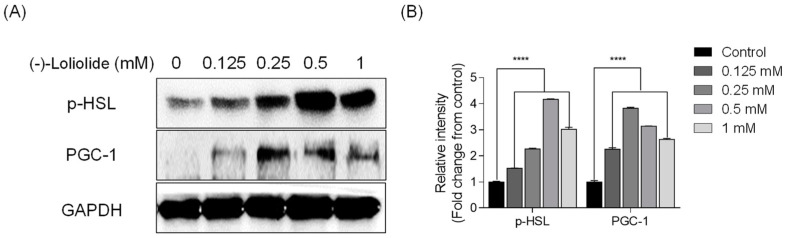
(−)-loliolide stimulates the expression of lipolytic and thermogenic proteins in 3T3-L1 cells. (**A**) Western blots showing expression of lipolytic protein p-HSL and thermogenic protein PGC-1. (**B**) Quantification graph for p-HSL and PGC-1 expressions. Significant differences were identified at **** *p* < 0.0001 compared to the control group.

Next, we assessed whether (−)-loliolide stimulates the expression of thermogenic peroxisome proliferator-activated receptor-γ coactivator-1α (PGC-1α) and lipolytic phospho-hormone sensitive lipase (p-HSL) in 3T3-L1 cells by Western blot analysis. As shown in Figure 3, the expression of PGC-1α and p-HSL, which was low in the control group, was considerably increased in (−)-loliolide-treated groups. These results suggest that (−)-loliolide from *S. horneri* could regulate lipolysis in vitro in 3T3-L1 cells.

## 3. Discussion

Obesity is regarded as a public health problem, and the overweight and obese populations are steadily increasing [33]. Most importantly, the prevalence of obesity-related complications has also grown in the past few decades [34,35,36]. Epidemiological obesity studies have revealed that overweight and obese individuals worldwide account for 33% of the total population [37]. Numerous anti-obesity mechanisms regulate lipid metabolism in obese patients. Among these, the inhibition of lipid accumulation is one of the best strategies to improve and treat obesity. According to in vitro and in vivo studies, inhibition of adipogenic and lipolytic protein expression is one of the possible approaches to suppress lipid metabolism [38]. However, relatively low inhibitory effects of marine natural products on lipid metabolism were reported compared to those from land plants. Hypolipidemic effects of marine-derived polysaccharides have been reported [39,40,41,42]. Nevertheless, few anti-obesity studies have been published on marine peptides, polyphenolic compounds, and pigments [23,43,44,45]. Therefore, to fill this knowledge gap, we investigated the potential inhibitory effect of the phenolic compound (−)-loliolide isolated from *S. horneri* on lipid accumulation in 3T3-L1 cells.

Intracellular lipid accumulation is a normal process in the human body. However, high-calorie food intake or overnutrition can cause excessive lipid accumulation in the adipocytes, resulting in obesity and excess weight. Normally, intracellular lipid accumulation in adipocytes is controlled by adipose-specific proteins, including lipogenic protein SREBP-1 and adipogenic proteins PPAR-γ, C/EBP-α, and FABP4. Adipogenic and lipogenic proteins play a crucial role in lipid metabolism, including lipid storage and synthesis in 3T3-L1 cells [46]. Sterol regulatory element-binding proteins (SREBPs), including SREBP-1a, SREBP-1c, and SREBP-2, are transcription factors. SREBP-1c regulates lipid synthesis and sterol levels and acts in concert with carbohydrate response element-binding protein (ChREBP) to stimulate de novo lipogenesis (DNL), which induces lipid synthesis in the adipose and liver tissues [47]. PPAR-γ is regarded as the major adipogenic protein that regulates lipid and glucose metabolism. Adipogenesis starts with the expression of pro-adipogenic genes, including C/EBP-α, C/EBP-β, and C/EBP-γ, which stimulate the expression of the key adipogenic protein PPAR-γ [48,49]. FABP4 is a lipid-trafficking protein controlled by PPAR-γ that plays a pivotal role in the transport of lipids from the extracellular to the intracellular matrix in adipocytes [50]. According to Kim et al. (1998), the expression of SREBP-1 specifically increases PPAR-γ activation by increasing endogenous ligands; moreover, adipose differentiation is controlled by interactions of transcriptional activation of PPAR-γ, C/EBPs, and SREBP-1 [51]. In addition, Horton et al. (2003) also reported that SREBP-1 expression affects the gene expression of PPAR-γ and C/EBP-α [52]. It has been suggested that adipogenesis is a mechanism that sustains adipocyte functions. p-HSL, known as TG lipase in adipose tissues, plays a critical role in maintaining energy homeostasis and mobilization of stored fat in the body [53]. PGC-1α is a transcriptional coactivator that regulates mitochondrial biogenesis by increasing the transcriptional activation of nuclear hormone receptors [54]. AMP-activated protein kinase (AMPK) plays an important role in sensing intracellular ATP levels and energy balance within the cells. According to Wan et al. (2014), AMPK regulates PGC-1α expression and mitochondrial enzymes in adipose tissues [55]. In addition, phosphorylation of AMPK activates lipolytic enzymes such as adipose TG lipase (ATGL) and HSL in white adipose tissues [56,57].

In this study, we evaluated the inhibitory effect of (−)-loliolide on lipid accumulation in differentiated 3T3-L1 cells and further investigated the effect of this compound on lipogenic, adipogenic, lipolytic, and thermogenic proteins in 3T3-L1 cells. We demonstrated the potential of (−)-loliolide to reduce expression of lipogenic protein SREBP-1 and adipogenic proteins PPAR-γ, C/EBP-α, and FABP4. These results are consistent with previous publications reporting hypolipidemic and anti-obesity activity of (−)-loliolide [58]. Furthermore, we found that the lipolytic p-HSL and thermogenic PGC-1α protein expressions were higher in 3T3-L1 cells treated with a low concentration of (−)-loliolide suggesting that the range of 0.125 to 0.25 μg/mL is a critical concentration.

Our findings indicate that (−)-loliolide significantly reduced lipid accumulation via regulation of adipogenic, lipolytic, and thermogenic proteins in 3T3-L1 cells.

## 4. Materials and Methods

### 4.1. Material and Reagents

The lipid staining dye Oil Red O (ORO, catalog: O0625), thiazolyl blue tetrazolium bromide (MTT), cell differentiation reagents 3-isobutyl-1-methylxanthine (IBMX, catalog: I7018), dexamethasone (DM, catalog: D8893), and insulin (catalog: I5500) were purchased from Sigma-Aldrich Co. (St. Louis, MO, USA). Dulbecco’s Modified Eagle’s Medium (DMEM, catalog: 12430-054) and supplements including bovine serum (BS, catalog: 26170-043), fetal bovine serum (FBS, catalog: 16000-044), penicillin/streptomycin (P/S, catalog: 15140-122), and trypsin-ethylenediaminetetraacetic acid (Trypsin-EDTA, catalog: 15400-054) were purchased from GIBCO-BRL (Grand Island, NY, USA). Isopropanol (2-propanol, catalog: 5035-44) was purchased from Daejung Chemicals & Materials (Siheung-si, Korea). Formalin (catalog: 6936050380) for cell fixation was purchased from Junsei (Tyoko, Japan). Primary antibodies against adipogenesis-related proteins, including PPAR-γ (catalog: #2443), C/EBP-α (catalog: #2295) and FABP4 (catalog: #2120), and against thermogenic proteins such as PGC-1α (catalog: #2178) and p-HSL (catalog: #4139), were purchased from Cell Signaling Technology (Bedford, MA, USA). Secondary antibody, anti-glyceraldehyde 3-phosphate dehydrogenase (GAPDH, catalog: sc-66163), and anti-SREBP-1 (catalog: sc-365513) were purchased from Santa Cruz Biotechnology (Santa Cruz, CA, USA).

### 4.2. Isolation and Purification of (−)-loliolide from S. horneri

The *S. horneri* was collected from the coastline of Jeju Island, Korea. The collected *S. horneri* was carefully washed using tap water and placed in a deep freezer for 24 h. Frozen *S. horneri* was lyophilized and ground using a grinder. A detailed purification method of (−)-loliolide was followed as previously described by Kim et al. (2020) [31]. Briefly, 100 g of the *S. horneri* powder samples was extracted under the optimal ethanol extraction conditions (37 °C, 24 h) in the shaking incubator. After 24 h of incubation, *S. horneri* 70% ethanol extracts (SHE) were filtered through Whatman No. 4 filter paper and dried under the vacuumed rotary evaporator using the volume flask. The dried samples were collected from the flask and lyophilized by freeze dryer (SFDSM06, SAMWON, Busan, Korea). The dried SHE was stored in the −80 °C freezer before use. The solvent fractionation was conducted on SHE. The dried SHE dissolved and fractionated with solvents (hexane, HX; chloroform, CHCl_3_; ethyl acetate, EA; and butanol, BtOH) sequentially. Among the solvent fractions, the CHCl_3_ fractions were collected from SHE and concentrated under the vacuum rotary evaporator. The partitioned CHCl_3_ was stored in the freezer at −80 °C for centrifugal partition chromatography (CPC). The CPC was adopted for isolate active compounds from SHE. The CPC column was equilibrated with the stationary phase and rotated at 1000 rpm. After the equilibration, the target sample was injected to the CPC device, and the mobile phase was eluted into the column in the descending mode at a flow rate of 2 mL/min. The CPC eluent was collected through fraction collector (FC 203B, Gilson, Villiers Le Bel, France). Then, high-performance liquid chromatography (HPLC) was conducted to further purify the active compound using a Prep HPLC system (Waters, Milford, MA, USA) equipped with a C18 (YMC-Pack, 5 µm, 10 × 250 mm), octadecyl-silica (ODS) column. The HPLC separation conditions were as follows: The gradient elution was carried out using acetonitrile and distilled water at 0–60 min, 5:95–100:0 *v/v*; and 60–70 min, 100:0–100:0 *v/v* at a flow rate of 3 mL/min. The UV absorbance was observed at 230 nm by a Waters 2998 photodiode array detector (Waters, Milford, MA, USA).

### 4.3. Cell Culture and Differentiation

The 3T3-L1 preadipocytes were purchased from the Korean cell line bank (KCLB). The cells were cultured in DMEM supplemented with 10% BS and 1% P/S under humidified conditions (5% CO2, 37 °C). The cells were subcultured every 2 days. To induce adipocyte differentiation, 3T3-L1 cells were plated in 12-well plates. Two days later, the cell confluence reached 100%, and the cell culture medium was changed to DMEM medium (containing 10% FBS and 1% P/S) with differentiation solutions (MDI) consisting of 0.5 mM IBMX, 0.25 μM DM, and 5 μg/mL insulin at day 2. Subsequently, the cell culture medium was switched to DMEM containing 10% FBS and 1 μg/mL insulin and maintained for 4 days to induce additional adipocyte differentiation. After day 4, the cell culture medium was replaced with fresh DMEM medium every 2 days. On day 8, 3T3-L1 cells were fixed and stained with 0.6% ORO solution to determine the accumulation of lipids in 3T3-L1 cells.

### 4.4. Cytotoxicity

The cytotoxic effect of (−)-loliolide was evaluated via MTT assay, as previously described by Lee et al. (2020) [59]. The 3T3-L1 cells treated with various concentrations of (−)-loliolide (0.125, 0.25, 0.5, and 1 mM) were incubated for 48 h in 48-well plates. After incubation, 75 μL of MTT solution (2 mg/mL) was added to each well and incubated in the dark for another 4 h. Subsequently, the well plates were centrifuged. After centrifugation, the supernatant was removed, and the precipitated formazan crystals, which were produced in the mitochondrial matrix of live cells, were dissolved in dimethyl sulfoxide (DMSO). To assess the cytotoxicity of (−)-loliolide, the formazan crystals were quantified and calculated using a microplate reader (Synergy™ HT Multi-Detection Microplate Reader, Bio-Tek, Winooski, VT, USA).

### 4.5. Oil Red O Staining

The specific lipid staining protocol was adopted to evaluate lipid accumulation in 3T3-L1 as previously described [60]. The 3T3-L1 cells were plated in 12-well plates, and adipocyte differentiation was induced with MDI solution for 8 days. During the adipocyte differentiation, the different concentrations of (−)-loliolide (0.062, 0.125, 0.25, 0.5, and 1 mM) were treated on days 0, 2, 4, and 6 except for the control group. On day 8, differentiated 3T3-L1 cells were fixed with 10% formalin for 1 h. Then, the fixed cells were washed twice with 60% 2-propanol and dried at 27 °C. The dried cell lipids were stained with 0.6% ORO solution for 2 h and again washed with distilled water (DW). After drying, ORO stain was eluted with 100% 2-propanol for 1 h in a shaking incubator. The relative ORO content was measured by Synergy^TM^ HT Multi-Detection Microplate Reader (Bio-Tek, Winooski, VT, USA). The images of the intracellular lipids from 3T3-L1 cells were captured using a microscope (Lionheart™ FX Automated Microscope, BioTek Instruments Inc., Winooski, VT, USA).

### 4.6. Western Blot Analysis

The effects of (−)-loliolide on the expression of adipose-specific proteins were investigated by Western blotting [61]. On day 8, differentiated 3T3-L1 cells were lysed in lysis buffer consisting of 5 mM EDTA, 10 mM Na4P2O7, 10 mg/mL leupeptin, 100 mM NaF, 1 mM PMSF, 20 mM Tris, 2 mM Na3VO4, 10 mg/mL aprotinin, and 1% NP-40. Protein concentration in lysates was determined by bicinchoninic acid assay (BCA protein assay kit, Thermo Fisher Scientific, Rockford, IL, USA). Equal amounts of proteins were loaded on 10–12% of sodium dodecyl sulfate (SDS) acrylamide gel and separated by electrophoresis. The electrophoresed samples were transferred onto nitrocellulose membranes. The transferred membranes were blocked in 5% skim milk for 2 h at room temperature. Then, the membranes were incubated with primary antibodies (1:1000) overnight at 4 °C. After incubation, the membranes were washed with 1 × tris-buffered saline (TBST) three times, followed by incubation with secondary antibodies (1:1000) for 2–3 h at room temperature. Finally, the protein bands were imaged by Fusion Solo imaging system (Vilber Lourmat, Paris, France) using the enhanced chemiluminescence (ECL) Western blot detection kit.

### 4.7. Statistical Analysis

All data are presented as mean ± standard deviation (SD), and experiments were performed at least three times. Statistical analysis was conducted using one-way ANOVA and Dunnett’s multiple comparisons test in Graph Pad Prism 6 software. The significant differences were expressed as follows: **** *p* < 0.0001.

## 5. Conclusions

In conclusion, the purified (−)-loliolide isolated from 70% ethanolic extract of *S. horneri* is a potential candidate for the development of a drug to inhibit lipid metabolism. (−)-loliolide may inhibit lipid metabolism by suppressing expression of adipogenic proteins and stimulating that of lipolytic and thermogenic proteins. However, large-scale observations are necessary to fully elucidate the inhibitory effect on lipid accumulation in detail. In conclusion, (−)-loliolide can be used as a valuable medicinal agent for the management of obesity.

## Data Availability

Not applicable.
